# Dark Septate Endophytic Fungi Improve Dry Matter Production and Fruit Yield in Ever-Bearing Strawberry (*Fragaria × ananassa* Duch.) Under High Temperatures

**DOI:** 10.3390/plants15010129

**Published:** 2026-01-02

**Authors:** Nanako Aomura, Ryuta Ninohei, Mana Noguchi, Midori Sakoda, Eiichi Inoue, Kazuhiko Narisawa, Yuya Mochizuki

**Affiliations:** College of Agriculture, Ibaraki University, 3-21-1 Chuo, Ami, Inashiki 300-0393, Ibaraki, Japanmana.noguchi.mesc@vc.ibaraki.ac.jp (M.N.); midori.sakoda.uh46@vc.ibaraki.ac.jp (M.S.); eiichi.inoue.a@vc.ibaraki.ac.jp (E.I.); kazuhiko.narisawa.kkm@vc.ibaraki.ac.jp (K.N.)

**Keywords:** DSE, ever-bearing strawberry, leaf emergence rate, light use efficiency, photosynthesis, bio stimulant, heat stress mitigation

## Abstract

In Japan, strawberries are produced in the off-season (June to November) in cool regions; however, the high temperatures and strong sunlight limit fruit production. Dark septate endophytic fungi (DSEs) support growth and flower bud formation of plants grown in environments unsuitable for plant growth. In this study, we investigated the effects of DSE on dry matter production and flower bud formation in strawberry plants grown in the summer and autumn. The seeds were sown in soil mixed with DSE on 5 February 2024. The DSEs used were *Cladophialophora chaetospira* SK51 (S) and *Cc*. MNB12 (M), and *Veronaeopsis simplex* Y34 (Y). Plants were planted in a plastic house on April 18. The total dry weight was significantly increased by DSEs. This is because S and Y-cultured plants did not show a significant decrease in leaf emergence under high temperatures, unlike those grown with M; however, its leaf area was larger than that of the control. This resulted in a larger leaf area for receiving light and higher cumulative light reception and light-use efficiency. Although the DSEs increased cumulative fruit yield, the harvest period was limited to July because of the extreme summer heat. In addition, there was no difference in the budding date or flowering date between the treatments. These results suggest that DSEs improve light use efficiency, thereby increasing total dry matter weight and contributing to increased fruit yield in summer-autumn cultivation.

## 1. Introduction

Strawberries (*Fragaria × ananassa* Duch.) are in high demand by consumers worldwide, regardless of the season, for uses ranging from raw consumption to processed products, such as jams and cakes. In Japan, 90% of fruit production is carried out between December and May through forced cultivation, using June-bearing cultivars [1]. Japanese high-quality strawberries are in high demand overseas, especially in Southeast Asia, and exports are increasing [2,3,4]. Therefore, there is a desire to increase year-round fruit production and production volume. Strawberries can be divided into June-bearing and Ever-bearing types based on the environmental conditions that induce flower bud differentiation. In June-bearing strawberries, flower bud differentiation is induced at low temperatures and on short days, whereas in ever-bearing strawberries, it is induced during long days [5]. Ever-bearing cultivars are grown in summer and autumn in Japan. However, high temperatures and strong light conditions decrease yields due to several factors, such as a decrease in plant vigor and inhibition of flower bud differentiation due to respiratory loss [6], small fruits due to a decrease in the number of achenes per fruit [7], and unfertilized fruits due to reduced pollen function [8]. To address these issues, producers have taken measures such as using covering materials, crown cooling, and fine mist cooling. Covering materials lower the temperature inside the greenhouse and provides shade, but limiting the light required for photosynthesis reduces the quantity of assimilation products [9]. Crown cooling improves commercial yields by suppressing the occurrence of infertile and small fruits but incurs running costs [10]. Furthermore, owing to recent extreme heat caused by global warming, new measures are needed to improve the physiological state of plants so that they can grow under high temperatures and strong light conditions.

Some microbes promote plant growth by coexisting with other plants. Rhizobia fix nitrogen from the atmosphere and supply it to plants, enabling legumes to grow under nutrient-poor conditions [11]. Arbuscular mycorrhizal fungi coexist with the roots and rhizosphere and promote the absorption of nutrients and water [12]. Dark septate endophytic fungi (DSE) are a class of fungi that form colonies by extending dark hyphae in the tissues and interior of roots without causing any visible symptoms in the host plant or establishing a symbiotic relationship [13]. By settling on the roots of plants, DSE supply nitrogen and phosphorus to the host plant and receive a carbon source from it [14]. Although rhizobia and other bacteria have specific host plants, DSE form symbiotic relationships with various plant species [13]. This suggests that DSE has low host specificity and can be used on a variety of plants. Moreover, DSE inoculation promotes plant growth under harsh environmental conditions such as high temperatures and drought, which are not suitable for plant growth. For example, studies have reported an increase in the antioxidant activity of medicinal plants cultivated under heat stress [15] and the pre-evolution of flower bud formation by 90 d in wild strawberries (*F. vesca*) cultivated under non-flower bud differentiation induction conditions [16]. In addition, effects such as solubilization of poorly soluble phosphate [17], reduction in heavy metal concentrations in plants [18], and suppression of soil-borne diseases [19,20] have been observed.

Increasing of fruti yield is caused by an increasing of total dry weight or dry matter distribution to the fruit. There are some reports that Increasing of fruits yield of tomatoes and strawberries is due to an increase in total dry weight [21,22,23]. In the winter-spring cultivation of strawberries, DSE promotes growth and accelerates the flowering of the first fruit cluster, thereby increasing yield [24]. This is thought to be because DSE improves the physiological conditions of plant growth, contributing to increased dry matter production and the promotion of flower bud formation. As mentioned above, the summer-autumn cultivation of strawberries in Japan is conducted under high-temperature and strong light conditions, which are not necessarily suitable for plant growth. Therefore, if strawberry growth can be supported by DSE inoculation, this will lead to increased yields during the off-season and the development of a new cropping type. Therefore, in this study, we investigated the effects of DSE inoculation on dry matter production and flower bud formation in summer-autumn strawberries using the seed-propagated strawberry cultivar ‘Yotsuboshi’, which possesses an ever-bearing trait.

## 2. Results

### 2.1. Dry Matter Production and Leaf Emergence Rate

The leaf and crown DWs of SK51 and MNB12 were significantly higher than those of C on November 5 (Table 1). The root and fruit DW of MNB12 was significantly higher than that of C on November 5. The petiole DW of SK51 was significantly higher than that of C on 5 November. Total DW was significantly greater in Y34 compared with that of C on August 22, and in SK51 and MNB12 than in C on November 5. No significant differences were observed in the total number of inflorescences among the treatments.

**Figure 1:**
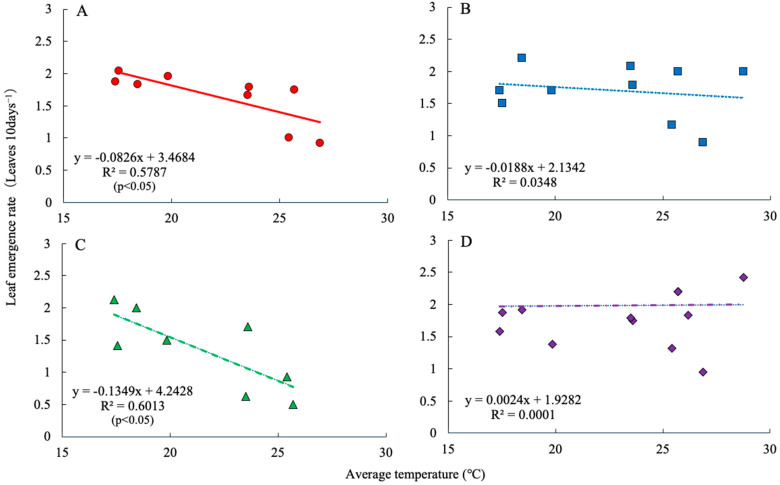
Relationship between average temperature and leaf emergence rate. Control (**A**), SK51 (**B**), MNB12 (**C**), Y34 (**D**).

There was a significant negative correlation between average temperature and leaf emergence rate in C and MNB12 (Figure 1A,C). In contrast, no significant correlation was observed between temperature and leaf emergence rate in the SK51 and Y34 plants (Figure 1B,D). 

### 2.2. Date of Flower Bud Emergence, Flowering, and Fruit Yield

No significant differences in flower bud emergence or flowering date were observed between the treatments (Table 2). The first and second inflorescences budded in mid-and early July, respectively. The cumulative fruit yield of C was 346 g m^−2^, while that of the DSEs increased to 441–462 g m^−2^ (Table 3). The fruit yields of SK51 and MNB12 were significantly higher than those of C. However, there were no significant differences in the monthly yield or fruit number between the treatments.

### 2.3. Projected Leaf Area (PLA), Cumulative Light Interception, and Light Use Efficiency (LUE)

**Figure 2:**
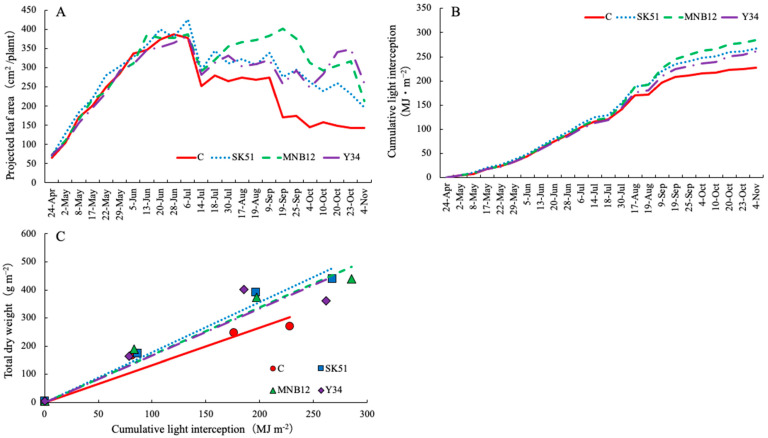
Changes in projected leaf area (**A**), cumulative light interception (**B**) and comparison of light use efficiency (**C**). Increase in dry weight after transplanting.

The rate of increase in the projected leaf area showed the same tendency among the treatments until June; however, the projected leaf area of DSEs tended to be higher than that of C from July (Figure 2A). The cumulative light interception at the end of the cultivation was 227.6 MJ m^−2^ in the C treatment, 267.3 MJ m^−2^ in the SK51, 285.0 MJ m^−2^ in the MNB12, and 261.0 MJ m^−2^ in the Y34, which were higher in the DSEs (Figure 2B). The light use efficiency calculated from the correlation between the total dry weight and the cumulative light interception was 1.33 g MJ^−1^ in C, 1.78 g MJ^−1^ in SK51, 1.69 g MJ^−1^ in MNB12, and 1.67 g MJ^−1^ in Y34; therefore, they were higher in the DSEs (Figure 2C).

### 2.4. Root Bleeding Rate and Nitrogen/Carbon Partitioning

**Figure 3:**
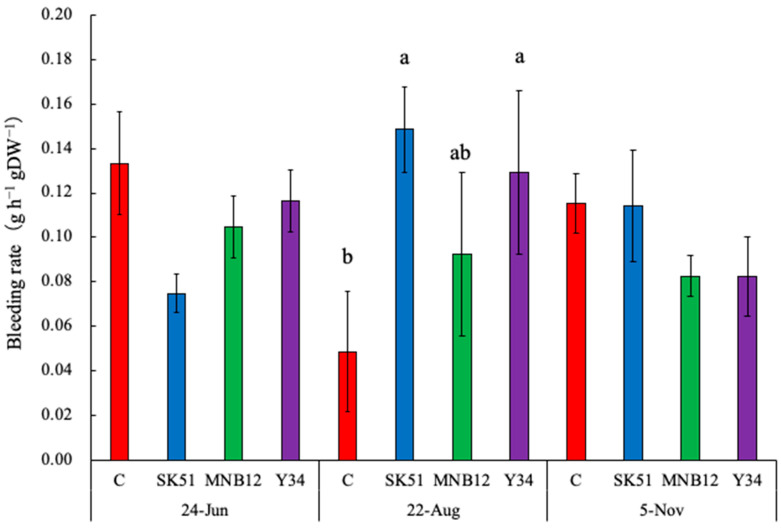
Comparison of Bleeding rate per root dry weight. Different letters represent significantly different values (*p* < 0.05; Tukey–Kramer test; n = 3–6).

No significant difference in the bleeding rate was observed between the treatments in the surveys conducted on 24 June and 5 November (Figure 3). However, in the extremely hot environment on 22 August, the bleeding rates of SK51 and Y34 were significantly higher than those of C.

Regarding the allocation of nitrogen and carbon, the carbon content in the leaves of Y34 was higher than that of MNB12, and the nitrogen content in the roots of MNB12 was higher than that of Y34 on 24 June; however, no other significant differences were observed (Table 4).

### 2.5. Diurnal Changes in Photosynthetic Rate and Maximum Quantum Yield of Photosystem II (Fv/Fm)

**Figure 4:**
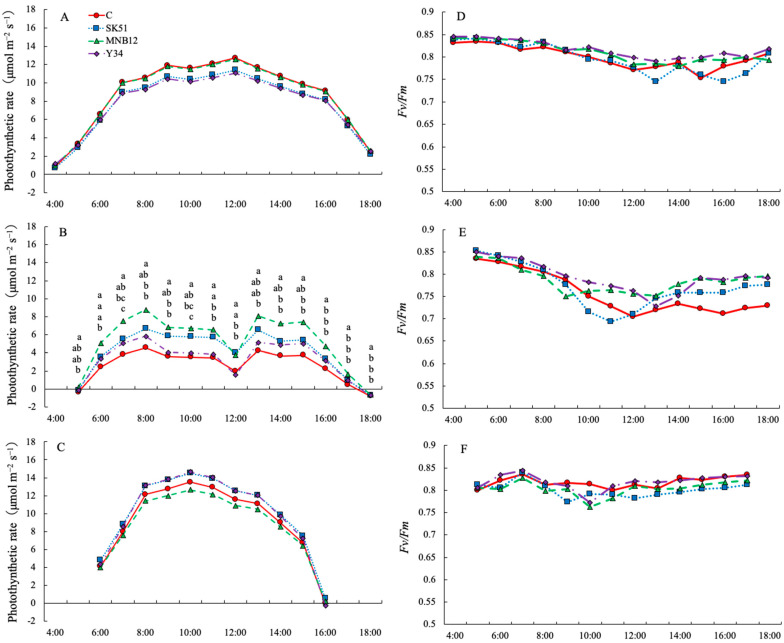
Diurnal changes in photosynthetic rate (**A**–**C**) and maximum quantum yield of photosystem II (Fv/Fm) (**D**–**F**) in July (**A**,**D**), August (**B**,**E**) and October (**C**,**F**). Different letters represent significantly different values (*p* < 0.05; Tukey–Kramer test; n = 3).

The diurnal changes in the photosynthetic rate in June and October showed a similar tendency; that is, they increased with sunrise and reached a maximum between 10:00 and 12:00. No significant differences were observed between the treatments (Figure 4A,C). However, the diurnal change in the photosynthetic rate in August was considerably lower than that in June and October and showed a different trend (Figure 4B). The rate was lower from 11:00 to 12:00, when the light intensity was high, compared with that under the active photosynthetic period from 7:00 to 16:00. This trend was similar across the treatments. However, the photosynthetic rates of MNB12 and SK51 were significantly higher than that of C.

Diurnal changes in Fv/Fm showed the same tendency as those in the photosynthetic rate. The value remained stable between 0.75 and 0.85 in June and October (Figure 4D,F), but decreased to 0.70 between 10:00 and 14:00 in August (Figure 4E). However, no significant differences were observed between the treatments.

## 3. Discussion

Total DW increased significantly in August and November, and the DW of each part increased significantly in November in the DSEs. DSEs increase aboveground DW in rice under drought conditions [25] and in Astragalus membranaceus under high temperatures [26], which is consistent with the results of the present study. The leaf expansion rate of tomatoes increases by 0.3–0.4 leaves per day for every 1 °C increase in the average temperature [27]. In the present study, the leaf emergence rates of C and MNB12 significantly decreased with increasing average temperature; however, this tendency was not observed in SK51 and Y34 (Figure 1). The leaf emergence rates of SK51 and Y34 did not decrease even during the high-temperature period, and the projected leaf area was secured; therefore, it is thought that the photosynthetic rate of the entire plant was higher than that of C. In contrast, the projected leaf area of MNB12 was larger than that of C (Figure 2A). This might be because the leaf emergence rate decreased because of high temperatures, but the leaf elongation rate was high, and the leaf area of individual leaves was larger than that of C. Matsubara et al. [28], who reported that when arbuscular mycorrhizal fungi were inoculated into strawberry seedlings and the seedlings were grown in a high-temperature environment, the leaf area and aboveground DW increased. In addition, the total fruit yield was higher in SK51 and MNB12 than in C. This is consistent with a report that the cumulative fruit yield of strawberries increased with DSE inoculation in winter-spring strawberry cultivation [24]. Kaneko et al. [29] and Mochizuki et al. [30] reported that to increase the fresh fruit yield of tomatoes and strawberries, it is important to ensure a sufficient LAI and increase the cumulative amount of light interception. In this study, cumulative light interception and light-use efficiency were greater in plants cultured with DSEs (Figure 2B,C). The contributing factor for total dry matter production is light-use efficiency, which is the amount of light received per area [31]. Improved light-use efficiency is known to increase tomato yield [21]. In this experiment, DSEs increased the projected leaf area, thereby increasing light-use efficiency, which was thought to have contributed to an increase in total DW.

**Figure 5:**
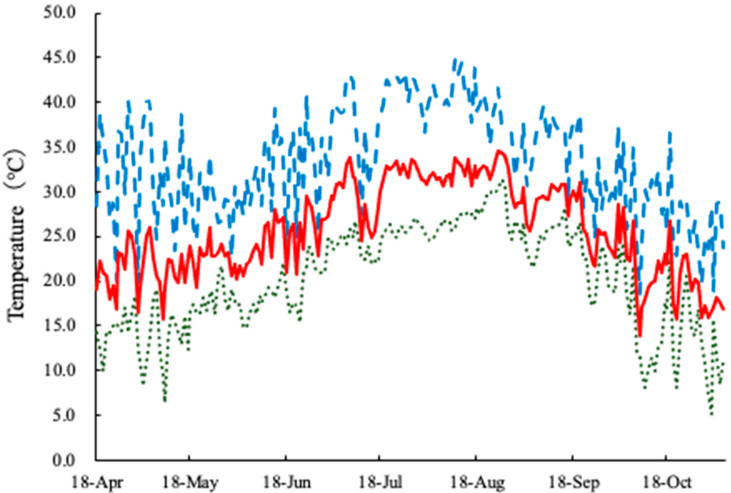
Maximum (blue dashed line), minimum (green dotted line), and average (red line) temperatures in the plastic house.

However, the fruit yield was low after August. The maximum temperature in the greenhouse exceeded 39 °C during July and August (Figure 5, Table 5), which is significantly higher than the optimum temperature for strawberry growth (15–25 °C) [1]. High temperatures increase the fluidity of membrane lipids, impede chloroplast photophosphorylation, inhibit photosynthesis, and cause protein denaturation [32]. We speculated that high temperatures significantly exceeding the optimum temperature for growth had a negative effect on the physiological state of the plant, causing some plants to die; therefore, sufficient yield could not be obtained after July. In addition, a previous report showed that SK51 promoted the flowering of *F. vesca* by 90 d [16], but we were unable to confirm the pre-flowering evolution by DSE in both the first and second inflorescences (Table 2). Hashiba et al. [33] reported that the establishment rate of endophytes decreased when sufficient nutrients were provided for plant growth. In this study, the establishment rate might be decreased owing to the application of liquid fertilizer adjusted to a concentration suitable for strawberry cultivation. Furthermore, no differences were observed in the rates of malformed fruits, unfertilized fruits, sugar content, or acidity between the treatments. Mori [7] reported that the lower the temperature during the pistil differentiation stage, the greater the number of achenes per fruit and the larger the fruit. Therefore, temperature during the pistil differentiation stage is an important factor to consider in the enlargement of strawberry fruits. In addition, Kawasato et al. [34] reported that stamens are easily affected by high temperatures during the flowering and tetrad formation stages, with the upper limit of high temperatures being approximately 40 °C. Based on these findings, it is necessary to combine growth promotion by DSE with flower bud induction by lowering the temperature around the plant, using methods such as mist or crown cooling.

The bleeding rates of SK51 and Y34 were higher in August than in C (Figure 3). Matsubara et al. [28] reported that inoculation with arbuscular mycorrhizal fungi suppresses the browning of strawberry roots grown at high temperatures. In this study, the dry weight of the roots of DSEs tended to be higher than that of C (Table 1), suggesting that the roots browned under high temperatures and root activity decreased. In addition, Mochizuki et al. [23,24] reported that high-yielding strawberry cultivars have high root volume and activity, and because they contain many ions, such as NO_3_^−^, large amounts of nitrogen can be distributed to each organ during root bleeding. In this study, it was speculated that the reason for the high yield of DSEs was that root volume and activity were maintained by mitigating high-temperature stress, and the DW of the aboveground parts was high. However, there were no significant differences in the nitrogen and carbon contents of each organ (Table 4), suggesting that increased water absorption might be important.

Inoculation with DSE improved the photosynthetic rate and maximum quantum yield of chlorophyll fluorescence in *Ammopiptanthus mongolicus* under water stress conditions [35]. In this study, the photosynthetic rate and Fv/Fm in August, when high-temperature stress was greatest, was lower than those in June and October (Figure 4). However, the photosynthetic rates of MNB12 and SK51 in August were higher than those of C, suggesting that photosynthetic function was maintained at a high level, improving light-use efficiency, and leading to an increased above-ground DW. Strawberry photosynthesis is known to be closely related to stomatal aperture and transpiration rate [36], and its effects have been reported to vary depending on light quality [37]. Therefore, DSE inoculation may have opened stomata even in high-temperature environments, promoting CO_2_ absorption and moderate transpiration, thereby maintaining high photosynthesis and increasing matter production. To further increase matter production during this period, increasing stomatal aperture and active CO_2_ uptake using LEDs might be effective.

## 4. Materials and Methods

### 4.1. Cultivation and DSE Inoculation

The strawberry cultivar ‘Yotsuboshi’, a seed-propagated strawberry, was used in this study. On 5 February 2024, 350 seeds from each treatment were sown in a cell tray (2.5 cm × 2.5 cm × 4.5 cm). There were four treatments: the control (C), no inoculation with DSE, and three inoculations with DSE. The DSE strains *Cladophialophora chaetospira* SK51 (SK51), *Cc*. MNB12 (MNB12), and *Veronaeopsis simplex* Y34 (Y34) were used in this study. SK51 was collected from root of *Spiranthes sinensis* at Shiga prefecture, Japan. MNB12 was collected from root of *Solanum melongena* at Shizuoka prefecture, Japan. Y34 was collected from root of *Solanum melongena* at Kagoshima prefecture, Japan. All the DSE strains were isolated at Ibaraki University. SK51 mitigates fusarium wilt disease and promotes plant growth and flowering [16,20,24]. MNB12 and Y34 mitigate heat stress under high temperatures [38,39,40]. Soil was prepared by mixing 5% DSE with sterilized organic soil (Meiko Co., Ltd., Hokkaido, Japan) adjusted to N: 80 mg L^−1^, P: 80 mg L^−1^, and K: 57 mg L^−1^. The DSE material was prepared by inoculating and culturing soil containing leaf mold, rice bran, and wheat bran in a 3:1:1 ratio. Primary nursing was conducted in an artificial climate room (Koito Electric Co., Ltd., Shizuoka, Japan). The environmental conditions were as follows: 16 h day length, 25 °C daytime temperature, and 20 °C nighttime temperature. The lighting source was an LED lamp (DLA-7T300, Daishin Co., Ltd., Hyogo, Japan), which provided a photosynthetic photon flux density of 130 μmol m^−2^ s^−1^. Water was provided twice a day in the morning and evening for up to one month after sowing. Subsequently, liquid fertilizer adjusted to pH 5.5–6.5 and EC 0.8 mS cm^−1^ was provided twice a day in the morning and evening. On 15 March 2024, the seedlings were transplanted into 24–hole trays (soil volume: 175 mL) filled with black peat moss (BVB soil; Toyotane Co., Ltd., Aichi, Japan), and secondary nursing was performed in a plastic house at Ibaraki University, Japan. Fertigation was prepared from OAT-A nutrient solution (OAT Agrio, Tokyo, Japan) with EC adjusted to 0.3–0.5 dS m^−2^ and pH of 6.5–7.0. The nutrient solution contained 2.2 to 3.7 mM NO_3_^−^, 1.0 to 1.7 mM K^+^, 1.0 to 1.7 mM Ca^2+^, 0.4 to 0.7 mM Mg^2+^, 0.6 to 1.0 mM H_2_PO_4_^−^, 0.3 to 0.5 mg L^−1^ Fe, 0.2 to 0.3 mg L^−1^ Mn, 0.2 to 0.3 mg L^−1^ B, 0.01 to 0.02 mg L^−1^ Zn, 0.003 to 0.005 mg L^−1^ Cu, and 0.003 to 0.005 mg L^−1^ Mo and was irrigated twice a day, in the morning and evening.

On 18 April 2024, six seedlings were planted in a polystyrene container (35.5 cm × 75.0 cm × 14.5 cm deep; 24 L) filled with BVB soil substrate. Seedlings were planted 30 cm apart, with 10 cm between rows. The plant density was 7.0 plants per m^2^. A total of 84 plants (across 14 containers) were randomly assigned to each treatment group. Plants were grown on a high bench in a plastic greenhouse at the College of Agriculture, Ibaraki University until 5 November 2024. The measurements of the plastic house were 5.5 m width, 27.5 m depth, and 3.5 m height. The height of the bench was 1.0 m. The plastic house was equipped with a circulation fan, and the top and side windows were left fully open for ventilation. Plants were irrigated OAT-A nutrient solution (OAT Agrio, Tokyo, Japan) with EC adjusted to 0.3–0.5 dS m^−2^ and pH to 6.5–7.0 via an automatic drip irrigation system (Aqua-touch; Sunhope-Aqua, Chiba, Japan) at approximately 30 mL per plant at 06:00 a.m. daily and again after every 2.0–2.5 MJ m^−2^ of cumulative solar radiation. The solar radiation sensors were placed in the plastic house. The buds were managed to ensure each plant had fewer than four. The senescent leaves were removed weekly. Artificial pollination was used to set up the fruits. The temperature inside the greenhouse was measured at 10 min intervals (Figure 1, Table 1) using a TR-76Ui CO_2_ Recorder (T&D, Tokyo, Japan). CO_2_ was supplied to the plants through the same porous tubes (WTR-100; Aomidori, Tokyo, Japan) placed within the canopy by a controller (Omnia Concerto, Tokyo, Japan) as described by Mochizuki et al. [41,42]. To induce flower bud initiation, plants were exposed to 24 h long-day lighting from the 1st to 14th of each month in July, August, and September. An LED light source (DLA-7T300, Daishin Co., Ltd., Hyogo, Japan) was placed 50 cm above the plant community and adjusted to provide a minimum of 1 μmol m^−2^ s^−1^ of light (1–10 μmol m^−2^ s^−1^).

### 4.2. Dry Matter Production and Leaf Emergence Rate

Destructive measurements were performed on April 19, June 24, August 22 and November 5. Three to 14 plants were harvested from each treatment and separated into roots, crowns, petioles, leaves, fruits, and peduncles, which were dried at 80 °C for 72 h in a circulation drier, cooled, and weighed to measure dry weight (DW). Leaves from destructively measured plants were obtained to measure the total leaf area. The leaves were photographed, and the leaf area was calculated using lia32 leaf area analysis software (https://www.flatworld.jp/soft/142711.html, accessed on 12 December 2025). The leaf emergence rate was calculated from the number of leaves and temperature data measured during weekly growth surveys.

### 4.3. Date of Flower Bud Emergence, Flowering, and Fruit Yield

The dates of bud emergence and flowering of the first flower on the first and second inflorescences of the main stem were recorded every two days.

Fruits with ≥80% skin color were harvested, and those with weights of >4 g were considered marketable fruits. The cumulative dry weight (DW) of the fruits was calculated as follows:

Cumulative fruit DW = (*FW*1 + *FW*2…*FWn*) · *r* (1)

where *FW*1, *FW*2, and *r* are the cumulative fresh weight of fruits harvested on the day of destructive measurement, the fresh weight of fruits that had been set on the day of destructive measurement, and the pre-measured dry matter ratio (0.127–0.135), respectively.

### 4.4. Projected Leaf Area (PLA), Cumulative Light Interception, and Light Use Efficiency (LUE)

The upper part of the plant canopy was photographed using a smartphone from April 18 to November 4. After the leaves were extracted from the photographs using Microsoft PowerPoint software, the light-receiving leaf area was calculated using lia32 software (https://www.agr.nagoya-u.ac.jp/~shinkan/LIA32/, accessed on 12 December 2025). This value was used to calculate the projected leaf area. The daily PLA was estimated using linear interpolation. Daily light reception (MJ m^−2^ d^−1^) was calculated as follows (Monsi and Saeki 2005 [43]):

Daily light reception = *DSR_total_* · *C_PAR_* · *LT_plastic house_* · *PLA* · *PD* (2)

where *DSR_total_*, *C_PAR_*, *LT_plastic house_*, *PLA*, and *PD* are the total daily solar radiation (data for Tsukuba, Ibaraki, Japan obtained from the Japan Meteorological Agency website were used, https://www.jma.go.jp/jma/indexe.html, accessed on 12 December 2025), photosynthetically active radiation coefficient (0.5), plastic house light transmittance (0.8), daily PLA, and planting density (7.0 m^−2^), respectively. Cumulative light interception was evaluated by measuring the daily light reception. LUE is the slope of the regression line between cumulative light interception and total DW.

### 4.5. Root Bleeding Rate and Nitrogen/Carbon Partitioning

The bleeding rate was measured based on the method of Mochizuki et al. [22]. Each plant was cut just above the junction of the shoot apex and the root system, and cotton was immediately placed on the cut surface and covered with a plastic film to eliminate the effects of transpiration. After two hours, the weight of the cotton that had absorbed the bleeding sap was measured, and the weight of the cotton used was subtracted to calculate the amount of bleeding per unit time. The plants whose bleeding rates had been measured were then pulled out, the roots were collected, and the amount of bleeding per gram of dry root weight was calculated. The bleeding rate was measured between 8:00 a.m. and 10:00 a.m.

To measure nitrogen and carbon contents, the leaves, crowns, and roots dried during the destructive investigation were ground into a powder using a grinder, and approximately 100 mg of each sample was analyzed using an NC analyzer (JM3000CN, J–Science Group, Kyoto, Japan). The carbon and nitrogen content of each organ was measured using three to five samples from each treatment. The helium and oxygen gas pressures were 0.4 MPa, respectively.

### 4.6. Diurnal Changes in Photosynthetic Rate and Maximum Quantum Yield of Photosystem II (Fv/Fm)

On 25 to 27 June, 10 to 13 August, 30 to 31 October, and 1 November, the photosynthetic rate (Pn) was measured using a portable photosynthesis system (LI−6800XT; LI−COR, Lincoln, NE, USA). Measurements were conducted between 04:00 and 18:00. The third fully expanded leaf of three plants in each treatment was used for the measurements. Photosynthetic characteristics were measured under basal conditions of 75 ± 10% humidity, leaf temperature of 25 °C, 400 µmol mol^−1^ CO_2_, and 600 µmol s^−1^ air flow. Light intensity was measured as the instantaneous value (μmol m^−2^ s^−1^) of direct sunlight outdoors on the day of measurement.

Diurnal changes in Fv/Fm were measured once a month (29 June, 25 August, and October 15) every hour from sunrise to sunset. Five to six plants from each treatment were used, and the leaflet located in the center of the fully expanded third leaf was treated with a special clip for 30 min in the dark before measurements were taken. A portable chlorophyll fluorescence meter (FluorPen FP110; PSI (Photon Systems Instruments), Drásov, Czech Republic) was used for measurements.

### 4.7. Statistical Analysis

All data were tested using one-way ANOVA followed by the Tukey–Kramer test. All statistical analyses were performed using JASP statistical software (https://jasp-stats.org/, accessed on 12 December 2025).

## 5. Conclusions

This study demonstrated that inoculation with DSE enhanced dry matter production and fruit yield in ever-bearing strawberries cultivated under high-temperature conditions. Specifically, the SK51 and MNB12 strains increased the total dry weight and cumulative fruit yield by improving the projected leaf area and light use efficiency. Furthermore, DSEs maintained root activity and partially preserved photosynthetic function during periods of extreme summer temperatures. Although no significant effects on the flowering time or fruit quality were observed, DSE inoculation proved to be an effective strategy for improving plant growth and productivity under suboptimal environmental conditions. These findings suggest that DSEs may serve as promising bio-stimulants for summer-autumn strawberry production, contributing to sustainable off-season fruit cultivation in a warming climate.

## Figures and Tables

**Table 1:** Changes in dry weight and number of inflorescences.

Date	DSE	Leaf (gDW m^−2^)	Crown (gDW m^−2^)	Root (gDW m^−2^)	Peduncle (gDW m^−2^)	Fruit (gDW m^−2^)	Total (gDW m^−2^)	Inflorescence (No./Plant)
19 Apr.	C	2.28	0.12	1.01	ND	ND	3.41	ND
	SK51	2.78	0.13	1.20	ND	ND	4.11	ND
	MNB12	2.78	0.18	1.62	ND	ND	4.59	ND
	Y34	2.97	0.14	1.41	ND	ND	4.52	ND
24 Jun.	C	112.0	7.01	41.8	5.69	ND	166.4	1.00
	SK51	115.2	6.92	47.0	4.58	ND	173.7	1.00
	MNB12	128.9	8.02	47.3	4.60	ND	188.8	0.83
	Y34	102.6	6.94	48.8	4.78	0.5	163.6	1.25
22 Aug.	C	141.1	9.92	30.7	17.9	48.2	247.9 b	5.00
	SK51	233.9	15.8	39.6	33.7	69.4	392.5 ab	5.00
	MNB12	223.6	14.6	48.9	14.9	71.5	373.6 ab	4.33
	Y34	214.6	16.8	37.9	51.8	81.5	402.5 a	7.33
5 Nov.	C	179.2 c	14.1 b	18.2 b	11.8 b	110.5 b	333.8 b	3.30
	SK51	291.3 a	26.8 a	30.5 ab	24.1 a	157.0 ab	529.7 a	3.80
	MNB12	278.7 ab	26.3 a	39.0 a	21.9 ab	169.3 a	535.2 a	4.27
	Y34	229.6 abc	23.2 ab	29.9 ab	15.5 ab	146.5 ab	444.7 ab	3.50

Different letters indicate significant differences (*p* < 0.05; Tukey–Kramer test; n = 10).

**Table 2:** Effect of DSE inoculation on flower bud initiation (n = 18).

DSE	Emergence Date		Flowering Date	
	First	Second	First	Second
C	18 Jun.	6 Jul.	24 Jun.	11 Jul.
SK51	16 Jun.	8 Jul.	22 Jun.	11 Jul.
MNB12	15 Jun.	10 Jul.	21 Jun.	15 Jul.
Y34	18 Jun.	13 Jul.	24 Jun.	15 Jul.

**Table 3:** Effect of DSE inoculation on fruit yield and the number of fruits.

	DSE	Jul.	Aug.	Sep.	Oct.	Total
Yield (g m^−2^)	C	333.3	13.0	0.0	0.0	346.4 b
	SK51	424.4	32.2	1.6	3.8	461.9 a
	MNB12	434.3	23.9	0.2	1.4	459.8 a
	Y34	403.3	34.8	1.2	2.0	441.4 ab
No. fruit (fruit m^−2^)	C	67.1	6.4	0.3	0.0	73.8
	SK51	77.0	8.8	0.7	1.4	87.9
	MNB12	76.9	9.7	0.1	0.3	86.9
	Y34	66.1	14.3	0.4	0.7	81.5

Different letters indicate significant differences (*p* < 0.05; Tukey–Kramer test; n = 18).

**Table 4:** Effect of DSE inoculation on nitrogen and carbon partitioning of each organ.

		Leaf		Crown		Root	
Date	DSE	Nitrogen	Carbon	Nitrogen	Carbon	Nitrogen	Carbon
24 Jun.	C	1.84	46.36 ab	1.13	49.81	1.50 ab	48.39
	SK51	1.93	46.82 ab	1.11	50.80	1.36 ab	47.53
	MNB12	1.95	45.26 b	1.45	49.22	1.80 a	46.53
	Y34	2.07	47.96 a	1.30	49.36	1.25 b	46.64
22 Aug.	C	1.61	48.33	1.75	48.57	1.34	49.25
	SK51	2.05	46.39	1.73	48.65	1.85	46.99
	MNB12	1.83	47.10	1.35	48.54	1.62	48.45
	Y34	1.93	47.05	1.60	47.91	1.53	47.84
5 Nov.	C	1.89	48.70	1.31	50.23	1.22	53.06
	SK51	2.21	50.01	1.48	50.48	1.32	53.08
	MNB12	2.07	49.59	1.32	49.18	1.50	51.24
	Y34	1.91	49.22	1.29	50.65	1.12	52.46

Different letters indicate significant differences (*p* < 0.05; Tukey–Kramer test; n = 3–6).

**Table 5:** Maximum, minimum and average temperatures for each month during the growing season.

	Apr.	May	June	Jul.	Aug.	Sep.	Oct.
Max	32.5	30.1	32.2	39.0	39.5	33.9	27.6
Min	14.7	15.2	18.8	24.8	27.3	23.5	15.0
Ave	21.4	21.9	24.6	30.6	32.0	27.5	20.8

## Data Availability

The original contributions presented in this paper are included in this article; further inquiries can be directed to the corresponding author.
